# Parasympathetic reactivation after maximal CPET depends on exercise modality and resting vagal activity in healthy men

**DOI:** 10.1186/s40064-015-0882-1

**Published:** 2015-02-27

**Authors:** Felipe A Cunha, Adrian W Midgley, Thiago Gonçalves, Pedro P Soares, Paulo Farinatti

**Affiliations:** Medical Sciences Graduate Program, Faculty of Medical Sciences, University of Rio de Janeiro State, Rio de Janeiro, Brazil; Institute of Physical Education and Sports, Laboratory of Physical Activity and Health Promotion, University of Rio de Janeiro State, Rio de Janeiro, Brazil; Department of Sport and Physical Activity, Edge Hill University, Ormskirk, Lancashire, UK; Department of Physiology and Pharmacology, Fluminense Federal University, Niterói, Rio de Janeiro, Brazil; Physical Activity Sciences Graduate Program, Salgado de Oliveira University, Niterói, Brazil

**Keywords:** Autonomic nervous system, Heart rate recovery, Heart rate variability, Spectral analysis, Ergometry, Cardiopulmonary exercise testing

## Abstract

**Purpose:**

The main purpose of this study was to investigate parasympathetic reactivation of the heart [evaluated through heart rate recovery (HRR) and HR variability (HRV)] after maximal cardiopulmonary exercise testing (CPET) using three different exercise modalities.

**Methods:**

Twenty healthy men, aged 17 to 28 yr, performed three maximal CPETs (cycling, walking, and running) separated by 72 h and in a randomized, counter-balanced order. HRR was determined from the absolute differences between HR_peak_ and HR at 1–3 min after exercise. The root mean square of successive R-R differences calculated for consecutive 30-s windows (rMSSD_30s_) was calculated to assess the parasympathetic reactivation after maximal CPET.

**Results:**

Lower HR_peak_, VO_2peak_ and energy expenditure were observed after the cycling CPET than the walking and running CPETs (*P* < 0.001). Both HRR and rMSSD_30s_ were significantly greater during recovery from the cycling CPET compared to the walking and running CPETs (*P* < 0.001). Furthermore, Δ rMSSD (i.e. resting *minus* postexercise rMSSD every 30 s into the recovery period) was positively related to the resting high-frequency component (HF), rMSSD, and standard deviation of all normal R-R intervals (SDNN) (r_s_ = 0.89 to 0.98; *P* < 0.001), and negatively related to the resting low-frequency component (LF) and sympathovagal balance (LF:HF ratio) after all exercise conditions (r_s_ = −0.73 to −0.79 and −0.86 to −0.90, respectively; *P* < 0.001).

**Conclusions:**

These findings support that parasympathetic reactivation after maximal CPET (as assessed by HRR and rMSSD_30s_) depends on exercise modality and cardiac autonomic control at rest.

## Background

Heart rate recovery (HRR) and heart rate variability (HRV) have emerged as noninvasive physiological markers to evaluate cardiac autonomic nervous system activity. When measured after maximal cardiopulmonary exercise testing (CPET) they are considered as powerful independent predictors of mortality in healthy subjects and in various clinical populations (Buchheit and Gindre 2006; Cole et al. 1999; Kannankeril et al. 2004; Tsuji et al. 1996). The early HRR after exercise (typically HR assessed at the 1^st^ min of recovery) has traditionally been used as an index of vagal activity (Pierpont et al. 2000), since its drop is a function of parasympathetic reactivation, with sympathetic withdrawal becoming more prominent later in recovery (Arai et al. 1989; Imai et al. 1994). For instance, some studies have proposed the use of HRR as a measure of autonomic dysfunction (Cole et al. 1999; Jouven et al. 2005). Another method for evaluating the autonomic modulation of HR is through HRV, which reflects beat-to-beat changes in HR, expressing the sympathovagal interaction obtained from the variation of both instantaneous HR and R-R intervals within the cardiac cycle (Task Force 1996). Most recently, it has been shown that the effect of parasympathetic drive on HRR seems to be less evident as previously believed (Buchheit et al. 2007b). Moreover, the time-varying analysis of HRV during exercise recovery, using the root-mean-square of the successive normal sinus R-R interval difference calculated for consecutive 30-s windows (rMSSD_30s_) to capture the instantaneous level of parasympathetic reactivation, seems to be a better tool to reflect postexercise parasympathetic reactivation (Buchheit et al. 2007a, b; Goldberger et al. 2006).

Due to their importance as clinical prognostic markers, several studies have investigated HRR and HRV after CPET performed on cycle ergometer (Danieli et al. 2014; Gaibazzi et al. 2004; Goldberger et al. 2006; Jouven et al. 2005; Ng et al. 2009) and treadmill (Buchheit and Gindre 2006; Cole et al. 1999; Dupuy et al. 2012; Morshedi-Meibodi et al. 2002; Vivekananthan et al. 2003). However, the HRR and HRV after maximal CPET may vary according to whether exercise is performed on treadmill or cycle ergometer, since the physiological strain induced by treadmill exercise [represented as peak HR (HR_peak_) and peak VO_2_ (VO_2peak_)] seems to be significantly greater than in cycle ergometer (Abrantes et al. 2012; Hill et al. 2003; Jamison et al. 2010). Only two studies have directly investigated the role of exercise modality on HRR after CPET and found faster HRR after cycle ergometry compared to treadmill exercise (Maeder et al. 2009; Rahimi et al. 2006). However, neither of these studies adopted the rMSSD_30s_ index to investigate the parasympathetic reactivation after maximal CPETs. Therefore, the extent to which postexercise parasympathetic reactivation (as measured by the rMSSD_30s_ index) depends on the exercise modality, remains unclear and warrants further investigation.

Another important question that has yet to be elucidated is the association between cardiac vagal modulation at rest versus parasympathetic reactivation after maximal CPET, since some studies have shown that HRR is positively correlated to resting HRV indexes (Danieli et al. 2014; Evrengul et al. 2006; Nunan et al. 2010), while others failed to find any relationship (Bosquet et al. 2007; Javorka et al. 2002). To the best of our knowledge, however, no study has investigated the relationship between resting and postexercise HRV markers (e.g. as calculated by the rMSSD_30s_ index). Therefore, the follow question remains: when the cardiac vagal activity at rest is low, would be the postexercise parasympathetic reactivation also low or there would be some dissociation?

In brief, it is unclear to what extent the exercise modality may influence the acute responses of cardiovascular autonomic control, as reflected by HRR and HRV (i.e. rMSSD_30s_) markers. Since both cycle ergometer and treadmill exercise are frequently used in clinical exercise testing, data on the influence of exercise modality on the behavior of parasympathetic reactivation markers would be important for accurate risk prognosis. Moreover, it remains unclear the extent to which resting vagal activity of HR is related (or not) to parasympathetic vagal reactivation represented by a faster HRR and increased rMSSD_30s_.

Thus, the main purpose of the present study was to therefore investigate the effect of maximal CPET (performed using three different exercise modalities - cycling, walking, and running) and resting vagal activity on parasympathetic reactivation expressed by HRR and rMSSD_30s_, in healthy males. We hypothesized that postexercise parasympathetic reactivation would be dependent on either exercised modality or resting vagal activity of HR.

## Material and methods

### Participants

A group of 20 healthy men with the following characteristics volunteered for the study: mean (SD) age, 21 (3.3) yr; height, 175.1 (6.3) cm; body mass, 76.1 (11.2) kg; body mass index, 24.8 (2.7) kg/m^2^; and body fat, 10 (5) %. The participants were college students who volunteered for the study. The inclusion criteria were: a) no use of medication that might influence the cardiovascular or metabolic responses to exercise (e.g. appetite suppressant, antidepressant, antihypertensive, neuroleptics, antiarrhythmic and lithium); b) no smoking or use of ergogenic substances that could affect exercise performance; and c) absence of cardiovascular, respiratory, bone, muscle, or joint problems that could compromise the safety of physical exercise. All participants were classified as being at low risk for cardiovascular disease (ACSM 2009). The study gained approval from the University of Rio de Janeiro State (UERJ) ethics committee board and prior to the commencement of the study, participants were informed of the potential risks and discomforts, and subsequently gave written informed consent.

### Experimental design

Each subject visited the laboratory four times on four separate days to undertake the following procedures:

- Visit 1. Complete a pre-participation screening questionnaire for cardiovascular risk and a questionnaire to identify aspects related to physical activity, to perform anthropometric measurements, assessment of resting HRV, and familiarization with the test protocols and equipment. All participants had previous experience with treadmill and cycle exercise and none presented difficulty, or movement limitation.
- Visits 2–4. Perform three maximal CPETs (cycling, walking, and running), separated by 72 h and performed in a randomized, counter-balanced order. All tests were conducted at approximately the same time of day (between 07:00 and 11:00 a.m.) to negate any effects of circadian variation.

### Maximal cardiopulmonary exercise testing (CPET)

Walking and running CPET were performed on the same motorized treadmill (Inbramed™ Super ATL, Porto Alegre, RS, Brazil) and the cycling CPET was performed on a cycle ergometer (Cateye EC-1600, Cateye™, Tokyo, Japan). The participants were verbally encouraged to perform a maximal effort during each CPET. The work rate increments were individualized to elicit each subject’s limit of tolerance within 8–12 min. Initially, a non-exercise model developed to estimate the VO_2_ of a healthy population aged 19 to 80 years-old was applied (Matthews et al. 1999). Based upon the predicted maximal oxygen uptake (VO_2max_), the final work rate was calculated using the American College of Sports Medicine (ACSM) equations for either cycling, walking, or running (ACSM 2009). For the cycling test, the mean (SD) predicted final power was 345 (40) W, and 0 W and 30 W were used for the 3-min warm-up period and for the initial work rate of the incremental test, respectively. The mean work rate increment was 31 W. The cycling cadence was maintained at 55 revs · min^−1^ throughout the test.

The walking test was characterized by simultaneous changes in speed and slope. A 3-min warm-up period was performed at 5.0 km · h^−1^ and 0% grade. The initial and final treadmill speeds for the CPET were fixed at 4.0 and 6.0 km · h^−1^, respectively. The treadmill slopes for 60% and 100% of predicted VO_2max_ were then calculated, respectively, for the initial period [mean (SD) 19.5 (1.4) %] and for the final work rate [mean (SD) 22.3 (1.5) %]. The mean work rate increment was 0.22 km · h^−1^ for speed and 0.31%.min^−1^ for slope.

For the running test, the mean (SD) predicted final speed was 14.4 (0.8) km · h^−1^ and the work rates of 40% and 60% of the predicted VO_2max_ were then calculated, respectively, for the 3-min warm-up period [mean (SD) 5.8 (0.3) km · h^−1^] and for the initial test work rate [mean (SD) 8.6 (0.5) km · h^−1^]. The treadmill slope was set at 1% throughout the running test. The mean work rate increment was 0.6 km · h^−1^.

The tests were considered as maximal if the subjects satisfied at least three of the four following criteria: a) maximum voluntary exhaustion defined by attaining a 10 on the Borg CR-10 scale; b) 90% of the predicted HR_max_ [220 – age] or presence of a heart rate plateau (ΔHR between two consecutive work rates ≤ 4 beats · min^−1^); c) presence of a VO_2_plateau (ΔVO_2_ between two consecutive work rates of less than 2.1 mL⋅kg^−1^⋅min^−1^); d) maximal respiratory exchange ratio (RER_max_) > 1.10 (Howley et al. 1995).

Breath-by-breath pulmonary gas exchanges and minute ventilation were retrospectively time-averaged into 30 s bins. The 30-s time averages provided a good compromise between removing noise from the VO_2_ data while maintaining the underlying trend (Midgley et al. 2007). Prior to testing, the gas analyzers were calibrated according to the manufacturer’s instructions using a certified standard mixture of oxygen (17.01%) and carbon dioxide (5.00%), balanced with nitrogen (AGA™, Rio de Janeiro, RJ, Brazil). Flows and volumes of the pneumotacograph were calibrated with a syringe graduated for a 3 L capacity (Hans Rudolph™, Kansas, MO, USA). The ambient temperature during all testing ranged from 21°C to 23°C and relative humidity ranged from 55% to 70%.

### Assessment of HR and HRV

HR and HRV were recorded by a telemetric HR monitor (RS800cx, Polar™, Kempele, Finland). The R-R intervals were downloaded by Polar Precision Performance Software (Polar™, Kempele, Finland) and averaged for each 30-s window. The sampling frequency was 1000 Hz and signal artifacts were filtered out by the program by excluding R-R interval values with differences of more than 30% of the preceding R-R interval (Yamamoto et al. 1991). All the time series of R-R intervals exhibited low noise (i.e. rate of erroneous R-R intervals ≤5%). For spectral analysis time series, R-R intervals were processed by an automatic algorithm for artifact removal and were subsequently processed by Fast Fourier Transform (FFT) using Welch’s method and a Hanning window with 50% overlap, using a customized algorithm from a Matlab routine (Matlab 6.0, Mathworks Inc., USA). The beat-by-beat R-R interval series were then converted into equally spaced time series with 200 ms intervals using cubic spline interpolation (Task Force 1996).

Time-domain analysis consisted of measures of R-R intervals (average of all normal R-R intervals), SDNN (standard deviation of all normal R-R intervals), and rMSSD (square root of the sum of successive differences between adjacent normal R-R intervals squared). In the frequency-domain, the power spectrum density function was integrated in the two classical frequency bands, as follows: 1) low frequency band (LF: 0.04 to 0.15 Hz); and 2) high frequency band (HF: 0.15 to 0.40 Hz) (Task Force 1996). The HF was used as an index of vagal modulation, whereas LF was considered as representative of both sympathetic and parasympathetic nervous system influences (Cooley et al. 1998; Montano et al. 1998). The spectral values were expressed as absolute power (ms^2^) and normalized units (n.u.) (Pagani et al. 1986). The LF:HF ratio was adopted as a marker of sympathovagal balance.

### Resting HRV assessment

The subjects were instructed not to engage in any form of physical exercise in the previous 24 h, to abstain from alcohol, soft drinks and caffeine in the 8 h preceding the test and to fast for 3 h. In the laboratory, participants laid quietly for 10 min in a quiet room, kept at a relatively constant temperature (21 to 23°C), after which the HRV was measured for 20 min in the supine position. The last 10 min of data were recorded as the HRV at rest. The resting HRV was always measured at approximately the same time of the day, between 07:00 and 11:00 a.m.

### Determination of postexercise HRR and HRV

Within 5 s after CPET cessation, participants were placed in the supine position. The HRR was assessed from the absolute differences between HR_peak_ and the HR values at 1–3 min after exercise (Cole et al. 1999). Apart from expression of HRR as absolute values, the relative decline in HR (e.g. 1^st^ to 3^rd^ min) were also calculated (%HRR = HRR / HR_peak_ × 100). To assess parasympathetic reactivation in the first 3-min after the end of each CPET, a time domain HRV vagal index (i.e. rMSSD) was calculated sequentially at each 30-s of the recovery period (rMSSD_30s_) (Goldberger et al. 2006).

### Data analysis

Statistical analyses were completed using IBM SPSS Statistics 22 software (SPSS™ Inc., Chicago, IL USA). Descriptive sample statistics are reported as the mean and standard deviation (SD). Differences in the maximal physiological results and heart rate for the first 3 min of recovery from each of the three CPET (cycling, walking, and running) were analyzed using marginal models using the Mixed procedure. The rMSSD data during the first 3 min of recovery from each CPET were time-averaged into 30 s bins and differences between exercise modalities and across time were analyzed using factorial marginal models. The residuals for the rMSSD_30s_ marginal model were highly positively skewed, which was addressed using a log_10_ transformation of the observed data. Different covariance structures for the repeated measures residuals were assumed for the marginal models, and the best fitting covariance structure was identified as that with the lowest Hurvich and Tsai criterion value. Post hoc pairwise comparisons, with Sidak-adjusted *P* values, were used where there were significant main or interaction effects. The relationship between LF, HF and LF:HF ratio at rest versus VO_2peak_ and versus Δ rMSSD (i.e. resting *minus* postexercise rMSSD) every 30 s into the recovery period from each CPET were analyzed using the Spearman’s correlation coefficient. Two-tailed statistical significance for all null hypothesis tests was accepted as *P* ≤ 0.05.

## Results

Resting HRV indices are shown in Table 1. Table 2 shows the maximal physiological responses for the cycling, walking, and running CPET, and the heart rate and percentage change in heart rate at 1, 2, and 3 min into recovery from each CPET. Significant main effects were observed for VO_2peak_ (F = 11.6, *P* < 0.001), HR_peak_ (F = 15.3, *P* < 0.001), peak oxygen pulse (F = 8.4, *P* = 0.001), RER_max_ (F = 7.7, *P* = 0.002), energy expenditure (F = 7.4, *P* = 0.001) and HR at the first minute of recovery (F = 23.9, *P* < 0.001). The VO_2peak_, HR_peak_, peak oxygen pulse, and energy expenditure were significantly higher in the walking and running CPET compared to the cycling CPET. Mean RER_max_ was significantly higher only during treadmill walking vs. running (*P* < 0.001), while energy expenditure was significantly higher during running vs. walking (*P* = 0.001). Heart rate recovery was significantly faster for the cycling CPET than for the walking and running CPETs at 1 and 2 min into recovery, but no significant differences between exercise modalities were observed at 3 min into recovery.

**Table 1:** **Resting heart rate variability indices (N = 20)**

**Variables**	**Mean (SD)**
Frequency domain	
LF (ms^2^)	1708 (459)
HF (ms^2^)	2400 (1308)
LF (n.u.)	37 (10)
HF (n.u.)	44 (12)
LF:HF ratio	0.9 (0.5)
*Time domain*	
R-R interval (ms)	1013 (163)
SDNN (ms)	92 (39)
rMSSD (ms)	104 (30)

LF = low frequency component; HF = high frequency component; LF:HF ratio = sympathovagal balance; R-R interval = average of all normal R-R intervals; SDNN = standard deviation of all normal R-R intervals; rMSSD = square root of the sum of successive differences between adjacent normal R-R intervals squared.

**Table 2:** **Mean (SD) maximal physiological values achieved during each of the cardiopulmonary exercise tests (CPETs) and heart rate during the first 3 min of recovery from each CPET**

	**Exercise modality**						***P***		
	**Cycling**		**Walking**		**Running**		**Cycling vs. walking**	**Cycling vs. running**	**Walking vs. running**
VO_2peak_ (ml · kg^−1^ · min^−1^)	39.0 (7.1)		43.8 (4.6)		44.4 (5.0)		0.001	<0.001	NS
VO_2peak_ (L · min^−1^)	2.934 (0.471)		3.320 (0.481)		3.342 (0.342)		<0.001	<0.001	NS
EE during CPET (kcal)	105 (29)		113 (19)		141 (42)		0.023	<0.001	0.001
HR_peak_ (beats · min^−1^)	187 (10)		193 (9)		196 (9)		0.003	<0.001	NS
Oxygen pulse (beats · ml^−1^)	15.7 (2.8)		17.3 (2.7)		17.1 (2.2)		0.002	0.005	NS
V_Emax_ (L · min^−1^)	92.0 (16.3)		92.4 (12.6)		97.6 (12.1)		NS	NS	NS
RER_max_	1.12 (0.04)		1.15 (0.04)		1.10 (0.04)		NS	NS	0.001
HR recovery 1^st^ min (beats · min^−1^)	147 (11)		159 (13)		163 (12)		<0.001	<0.001	NS
HR recovery 2^nd^ min (beats · min^−1^)	117 (13)		126 (13)		128 (11)		0.001	<0.001	NS
HR recovery 3^rd^ min (beats · min^−1^)	108 (11)		112 (12)		114 (10)		NS	0.002	NS
Δ HR recovery 1^st^ min (%)	22 (3)		18 (4)		17 (4)		<0.001	<0.001	NS
Δ HR recovery 2^nd^ min (%)	37 (6)		35 (4)		35 (4)		0.046	0.013	NS
Δ HR recovery 3^rd^ min (%)	42 (5)		42 (4)		42 (4)		NS	NS	NS

VO_2peak_ = peak oxygen uptake; EE = energy expenditure; HR_peak_ = peak heart rate; V_Emax_ = maximal minute ventilation; RER_max_ = maximal respiratory exchange ratio; HR = heart rate; Δ HR = percentage difference between the peak heart rate and the heart rate at 1, 2, or 3 min into the recovery period; *P* = Sidak-adjusted *P* value; NS = not statistically significant.

**Figure 1:**
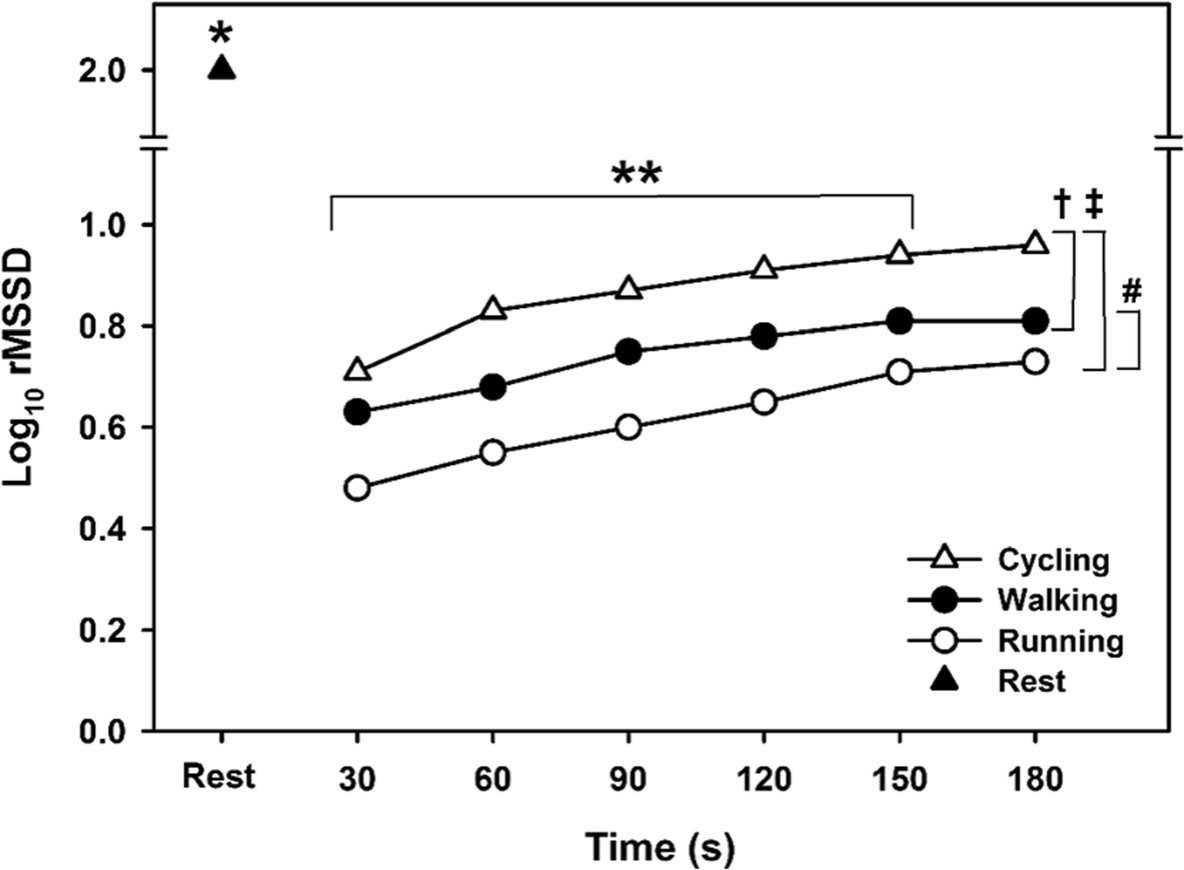
**Mean log**_**10**_ **rMSSD at rest and during the first 3 min of recovery from each cardiopulmonary exercise test.** Error bars have been omitted to aid clarity. * Resting log_10_ rMSSD significantly higher than each time point during the 3 min recovery period for each cardiopulmonary exercise test (P < 0.001); ** log_10_ rMSSD at each successive time point between 30 and 150 s significantly higher (*P* < 0.001); † cycling vs. walking (*P* < 0.001); ‡ cycling vs. running (*P* < 0.001); # walking vs. running (*P* < 0.001).

The rMSSD_30s_ significantly increased over the first 3 min of recovery from each CPET (F = 75.6, *P* < 0.001) (Figure 1). Post hoc pairwise comparisons showed that each successive time point was significantly higher than the previous time point (*P* ≤ 0.001), except for the difference between 150 and 180 s (*P* = 0.86). The rMSSD_30s_ response during the first 3 min of the recovery period was affected by exercise modality (F = 29.4, *P* < 0.001), where rMSSD_30s_ was significantly higher during the recovery from cycling compared to walking (*P* < 0.001) and running (*P* < 0.001), and significantly higher during recovery from walking compared to running (*P* < 0.001). No significant interaction between exercise modality and time was observed (F = 1.7, *P* = 0.10). The rMSSD_30s_ at 30 s of recovery was significantly lower than that at rest for each of the three CPETs (*P* < 0.001).

Table 3 presents the relationships between the LF, HF, LF:HF ratio, SDNN, and rMSSD at rest vs. ΔrMSSD (i.e. resting rMSSD *minus* postexercise rMSSD) every 30 s into the recovery period from each CPET. Significant negative relationships were observed between the LF and LF:HF ratio at rest and ΔrMSSD within 3 min of recovery from each CPET (r_s_ = −0.73 to −0.79 and −0.86 to −0.90, respectively; *P* < 0.001). The HF, SDNN and rMSSD at rest were positively correlated with ΔrMSSD, with Spearman’s correlation coefficients (r_s_) ranging from 0.90 to 0.98 (*P* < 0.001) for all CPETs.

**Table 3:** **Relationships between** Δ **rMSSD vs. LF at rest,** Δ **rMSSD vs. HF at rest,** Δ **rMSSD vs. LF:HF ratio,** Δ **rMSSD vs. rMSSD at rest, and** Δ **rMSSD vs. SDNN at rest every 30 s into the recovery period for each of the three exercise conditions (N = 20)**

**Relationships**	**Exercise modality**		**Spearman’s correlation coefficient (** ***P*** **value)**				
			**Exercise recovery time**				
		**30 s**	**60 s**	**90 s**	**120 s**	**150 s**	**180 s**
Δ rMSSD vs. rMSSD at rest	Cycling	0.92 (<0.001)	0.92 (<0.001)	0.91 (<0.001)	0.91 (<0.001)	0.91 (<0.001)	0.90 (<0.001)
	Walking	0.94 (<0.001)	0.94 (<0.001)	0.93 (<0.001)	0.93 (<0.001)	0.93 (<0.001)	0.92 (<0.001)
	Running	0.96 (<0.001)	0.96 (<0.001)	0.96 (<0.001)	0.94 (<0.001)	0.94 (<0.001)	0.92 (<0.001)
Δ rMSSD vs. SDNN at rest	Cycling	0.96 (<0.001)	0.96 (<0.001)	0.94 (<0.001)	0.91 (<0.001)	0.91 (<0.001)	0.90 (<0.001)
	Walking	0.96 (<0.001)	0.96 (<0.001)	0.94 (<0.001)	0.94 (<0.001)	0.94 (<0.001)	0.94 (<0.001)
	Running	0.98 (<0.001)	0.98 (<0.001)	0.98 (<0.001)	0.97 (<0.001)	0.97 (<0.001)	0.96 (<0.001)
Δ rMSSD vs. HF at rest	Cycling	0.92 (<0.001)	0.91 (<0.001)	0.91 (<0.001)	0.90 (<0.001)	0.89 (<0.001)	0.90 (<0.001)
	Walking	0.92 (<0.001)	0.91 (<0.001)	0.91 (<0.001)	0.91 (<0.001)	0.90 (<0.001)	0.92 (<0.001)
	Running	0.92 (<0.001)	0.91 (<0.001)	0.91 (<0.001)	0.93 (<0.001)	0.92 (<0.001)	0.91 (<0.001)
Δ rMSSD vs. LF at rest	Cycling	−0.79 (<0.001)	−0.77 (<0.001)	−0.77 (<0.001)	−0.74 (<0.001)	−0.73 (<0.001)	−0.74 (<0.001)
	Walking	−0.78 (<0.001)	−0.76 (<0.001)	−0.77 (<0.001)	−0.77 (<0.001)	−0.75 (<0.001)	−0.76 (<0.001)
	Running	−0.78 (<0.001)	−0.79 (<0.001)	−0.79 (<0.001)	−0.78 (<0.001)	−0.77 (<0.001)	−0.77 (<0.001)
Δ rMSSD vs. LF:HF ratio at rest	Cycling	−0.89 (<0.001)	−0.88 (<0.001)	−0.88 (<0.001)	−0.87 (<0.001)	−0.86 (<0.001)	−0.87 (<0.001)
	Walking	−0.88 (<0.001)	−0.87 (<0.001)	−0.88 (<0.001)	−0.87 (<0.001)	−0.87 (<0.001)	−0.88 (<0.001)
	Running	−0.89 (<0.001)	−0.90 (<0.001)	−0.89 (<0.001)	−0.90 (<0.001)	−0.89 (<0.001)	−0.88 (<0.001)

rMSSD = root-mean-square of the successive normal sinus R-R interval difference; SDNN = standard deviation of all normal R-R intervals; Delta (Δ) = resting rMSSD *minus* postexercise rMSSD; LF = high frequency component; HF = low frequency component; LF:HF ratio = sympathovagal balance.

## Discussion

The present study adds to current knowledge by investigating whether different exercise modalities (cycling, walking and running) and cardiac vagal activity at rest would influence parasympathetic reactivation after maximal CPETs in healthy young men. The major findings were: 1) Parasympathetic reactivation after maximal CPET (as assessed by HRR and rMSSD_30s_) seemed to be dependent on either exercised modality or cardiac autonomic control at rest. Actually the HRR and change in the rMSSD_30s_ index were faster for the cycling CPET (exercise involving a lower muscle mass) than for the walking and running CPETs (exercise involving a greater muscle mass); and 2) A mitigated HR response during exercise recovery was significantly correlated with a resting sympathetic activity overload (i.e. increased LF component and LF:HF ratio).

Rahimi et al. (2006) investigated the HRR from cycle ergometer and treadmill exercise in 211 individuals with known or suspected coronary artery disease. Although the treadmill exercise induced a higher HR_peak_, cycling presented a greater fall in HR during the early phase of recovery (~12% for treadmill vs. ~16% for cycle ergometer, *P* = 0.004). Similar findings were observed by Maeder et al. (2009) who compared the HRR after maximal CPETs performed on a treadmill and cycle ergometer in 29 healthy subjects and 16 patients with chronic heart failure. For both groups, HRR obtained in the first minute was significantly faster after cycling than after treadmill exercise (health and heart failure groups: 15% and 12% for treadmill vs. 17% and 15% for cycle ergometer, respectively; *P* = 0.004). In fact, our data showed that although both treadmill exercises (walking and running) induced a higher HR_peak and_ VO_2peak_, cycle ergometry resulted in a faster HRR only for the first minute of recovery (18% and 17% for walking and running CPETs vs. 22% for cycling CPET, *P* < 0.001), which in turn, are in agreement with the findings of previous studies (Maeder et al. 2009; Rahimi et al. 2006). Similar to HRR, the recovery of rMSSD_30s_ during the first 3 min after CPET cessation was higher for cycling vs. walking (*P* < 0.001) and running (*P* < 0.001); but unlike HRR – where no differences were observed between walking vs. running CPETs - the parasympathetic activation by rMSSD_30s_ index after walking CPET was significantly higher compared to running CPET (*P* < 0.001; see Figure 1). A possible explanation for these results is that HRR mainly describes the chronotropic response in absolute values and variations between beats at maximal HR and one value during recovery (i.e. reflecting a marker of parasympathetic tone), while rMSSD_30s_ is basically a measure of modulation based on adjustments on beat-by-beat dynamics (Buchheit et al. 2007b; Goldberger et al. 2006). Moreover, it is important to mention that although there was no significant difference between walking and running CPETs for HR_peak_ (*P* = 0.17) and VO_2peak_ (*P* = 0.90), the energy expenditure during running the CPET was significantly higher than that observed during walking CPET (*P* = 0.001; see Table 2).

Therefore, a question remains: what could explain the differences in postexercise parasympathetic reactivation between the three exercise modalities? Nowadays, it is widely accepted that HRR and HRV dynamics after exercise are affected mainly by exercise intensity (Lucini et al. 2014). In the present study, maximal effort was attained in all exercise modes, but for different absolute values of VO_2_ and HR to satisfy the energy demands of working muscles (see Table 2). These differences may have influenced the underlying mechanisms of cardiodeceleration after maximal CPETs (i.e. cycling, walking and running). Although the autonomic contribution to cardiodeceleration after exercise is less understood, passive recovery from dynamic exercise is associated with the cessation of the primary exercise stimulus from the brain (i.e. central command from the cerebral motor cortex), which seems to be responsible for the early recovery phase of HR (Carter et al. 1999). Likewise, changes in the stimuli to metaboreceptors and baroreceptors accompanying clearance of metabolites and neurohumoral factors (i.e. norepinephrine, epinephrine, angiotensin, endothelin and vasopressin), or lactic acid, may contribute to HRR or HRV dynamics after exercise (Kübler 1994). For instance, findings from Hambrecht et al. (1992) showed that the physiological strain induced by cycle ergometry were associated with 20% less norepinephrine and epinephrine levels at maximal effort than treadmill exercise, despite the lower HR_peak_ achieved. Furthermore, the circulating plasma catecholamines at 3 min of recovery from cycle ergometry were significantly lower than those induced by treadmill exercise (Hambrecht et al. 1992). Although we did not measure catecholamine kinetics after exercise, it is possible that the a slower removal of accumulated metabolites (i.e. higher catecholamine levels) after the running CPET has led to a blunted parasympathetic activity and reduced sympathetic withdrawal during the early phase of recovery, which may have resulted in a delayed HRR compared to cycling and walking CPETs. For example, Miyamoto et al. (2003) showed that high levels of norepinephrine attenuate the HR response to vagal stimulation by activation of the α-adrenergic receptors on the preganglionic and/or postganglionic cardiac vagal nerve terminals, leading to a reduced acetylcholine release in response to preganglionic vagal stimulation. Thus, our findings suggest that the effect of exercise modality on the postexercise reactivation should be taken into account for clinical applications, particularly with regard to the HR reduction during the first 30 s of recovery, which seems to be vagally mediated (Imai et al. 1994).

Another important finding of the present study is the influence of cardiac autonomic control at rest on the recovery pattern of HR after maximal CPET. Although the few available studies regarding the relationship between resting cardiac parasympathetic control and HRR have shown conflicting results (Bosquet et al. 2007; Danieli et al. 2014; Esco et al. 2010; Javorka et al. 2002; Nunan et al. 2010), none of these studies investigated the relationship between resting and postexercise HRV indexes. For instance, the recent findings of Duarte et al. (2014) provided evidence that adaptations to aerobic training in cardiac autonomic control at rest and during recovery from exercise present distinct dynamics. In this study, young subjects with lower and higher cardiac vagal modulation at rest underwent 12 weeks of aerobic training, and HRV at rest (i.e. HF, LF and LF:HF ratio) and parasympathetic reactivation (i.e. rMSSD_30s_) were assessed using the same approach as in the present paper. These authors showed that subjects with lower pre-training resting HRV indices (e.g. **↓** HF and **↑** LF or LF:HF ratio, respectively) improved their autonomic profile while the ones with previous elevated vagal modulation did not. Moreover, the relationship between resting vagal modulation and postexercise vagal reactivation was observed only in subjects with baseline low levels of resting vagal control (e.g. correlation between Δ% HF vs. Δ% rMSSD_3-5min,_ r = 0.63; *P* = 0.04). On the other hand, both groups improved HRR, suggesting that the mechanisms involved in HR dynamics at rest and after exercise may not act in the same way. Resting HR oscillations depend mainly on sympathetic and vagal modulatory influences, but after maximal exercise, when parasympathetic contribution is negligible, the fast recovery is dependent on vagal reactivation with a later influence of both vagal reactivation and sympathetic withdrawal. Moreover, vagal recovery is improved with greater availability of acetylcholine by anticholinesterase inhibitor administration (Dewland et al. 2007). These are different situations, although variability still primarily depends on vagal contribution, regardless of the exercise modality. For example, Table 3 shows that all HRV indexes from spectral analysis (i.e. frequency and time domain) were significantly and strongly correlated with ΔrMSSD at each 30 s interval during recovery. The resting parasympathetic activity (HF component), which reflects the magnitude of the fluctuation in cardiac vagal activity (Hedman et al. 1995), was positively related to ΔrMSSD_30-180s_ (i.e. r_s_ ranging from −0.90 to −0.93; *P* < 0.001). On the other hand, the LF component, influenced by both sympathetic and parasympathetic discharges, and the sympathovagal balance (LF:HF ratio) were negatively related to ΔrMSSD_30-180s_ (r_s_ = −0.73 to −0.79 and −0.86 to −0.90, respectively; *P* < 0.001). Indeed, it has been suggested that a mitigated HR response to exercise is indicative of a resting sympathetic overload (Colucci et al. 1989). Nevertheless, it is noteworthy that the utility of the LF band and LF:HF ratio as only a marker of cardiac sympathetic outflow is debatable, regardless of adjustment for total power, suggesting that the LF component is also determined by cardiac parasympathetic tone (Reyes del Paso et al. 2013). However, during maneuvers that increase the adrenergic drive (i.e. like exercise stress), usually a reduction of the HF and an increase in LF is observed, suggesting a shift in the autonomic balance that may induce the concept of a predominant sympathetic origin of the LF component (Cooley et al. 1998; Montano et al. 1994, 1998). In addition, HRR seems to be dependent on vagal reactivation after almost total withdrawal during heavy exercise, a phenomenon distinct from vagal modulation at rest when both branches of the autonomic nervous system discharge provide the oscillatory nature of HR (Dewland et al. 2007; Duarte et al. 2014; Goldberger et al. 2006). Although the present findings should therefore be interpreted with caution and further experimental research is warranted to confirm this hypothesis, our data seems to support that postexercise vagal reactivation seems to be dependent on the resting cardiac vagal control (i.e. higher resting vagal modulation would result in a better chronotropic response after maximal CPET). In a clinical context, these findings have direct implications on the interpretation of the influence of exercise modality and resting vagal modulation upon HRR.

## Conclusion

In conclusion, postexercise vagal reactivation (as measured by HRR and, mainly, by rMSSD_30s_) was shown to be faster after exercise involving smaller muscle mass or energy expenditure (cycling > walking > running) in healthy young men and this information should be considered in clinical settings. Moreover, postexercise parasympathetic reactivation seems to be influenced by resting vagal control, whereby subjects characterized by higher vagal modulation at rest tend to exhibit better parasympathetic reactivation and faster HRR. In a practical perspective, the resting vagal modulation appears to play a key role in postexercise parasympathetic activation, regardless of the exercise modality. These findings have direct implications on the interpretation of the influence of exercise modality and resting vagal modulation upon HRR.

## Abbreviations

CPET: Cardiopulmonary exercise testing
EE: Energy expenditure
HF: Low frequency component
HR: Heart rate
HR_peak_: Peak heart rate
HRR: Heart rate recovery
HRV: Heart rate variability
LF: High frequency component
LF:HF ratio: Sympathovagal balance
RER_max_: Maximal respiratory exchange ratio
rMSSD: Root-mean-square of the successive normal sinus R-R interval difference
rMSSD_30s_: Root mean square of successive R-R differences calculated for consecutive 30-s windows
SD: Standard deviation
SDNN: Standard deviation of all normal R-R intervals
V_Emax_: Maximal minute ventilation
VO_2peak_: Peak oxygen uptake
Δ rMSSD: Resting rMSSD *minus* postexercise rMSSD
